# Solution‐Processed Bilayer Dielectrics for Flexible Low‐Voltage Organic Field‐Effect Transistors in Pressure‐Sensing Applications

**DOI:** 10.1002/advs.201701041

**Published:** 2018-07-11

**Authors:** Zhigang Yin, Ming‐Jie Yin, Ziyang Liu, Yangxi Zhang, A. Ping Zhang, Qingdong Zheng

**Affiliations:** ^1^ State Key Laboratory of Structural Chemistry Fujian Institute of Research on the Structure of Matter Chinese Academy of Sciences 155 Yangqiao Road West Fuzhou Fujian 350002 China; ^2^ Photonics Research Center Department of Electrical Engineering The Hong Kong Polytechnic University Hong Kong SAR China

**Keywords:** dielectrics, flexible electronics, organic field‐effect transistors, pressure sensitivity, pressure sensors

## Abstract

Flexible pressure sensors based on organic field‐effect transistors (OFETs) have emerged as promising candidates for electronic‐skin applications. However, it remains a challenge to achieve low operating voltages of hysteresis‐free flexible pressure sensors. Interface engineering of polymer dielectrics is a feasible strategy toward sensitive pressure sensors based on low‐voltage OFETs. Here, a novel type of solution‐processed bilayer dielectrics is developed by combining a thick polyelectrolyte layer of polyacrylic acid (PAA) with a thin poly(methyl methacrylate) (PMMA) layer. This bilayer dielectric can provide a vertical phase separation structure from hydrophilic interface to hydrophobic interface which adjoins well to organic semiconductors, leading to improved stability and remarkably reduced leakage currents. Consequently, OFETs using the PMMA/PAA dielectrics reveal greatly suppressed hysteresis and improved mobility compared to those with a pure PAA dielectric. Using the optimized PMMA/PAA dielectric, flexible OFET‐based pressure sensors that show a record high sensitivity of 56.15 kPa^−1^ at a low operating voltage of −5 V, a fast response time of less than 20 ms, and good flexibility are further demonstrated. The salient features of high capacitance, good dielectric performance, and excellent reliability of the bilayer dielectrics promise a bright future of flexible sensors based on low‐voltage OFETs for wearable electronic applications.

## 1 Introduction

Over the past decade, electronic skin (e‐skin) has been developed as a viable technology to mimic temperature, humidity, pressure, and strain sensing of human skin.^1^, ^2^, ^3^ As a basic part of e‐skin, pressure sensors are attracting growing attention because they can detect tiny pressure change by converting an external force to electrical or other recognized signals.^4^, ^5^ This function makes them great potentials in wearable electronics, biomedical diagnostics, health monitoring, and so forth.^6^, ^7^, ^8^, ^9^ So far, various transduction methods such as piezoresistivity, capacitance, and piezoelectricity have been used for different types of pressure sensors and their integrated devices.^6^, ^10^, ^11^, ^12^ Among them, transistor‐based sensors usually perform pressure detection via a capacitance mechanism, where a tiny change in capacitance under tactile stimuli will produce a large current signal.^13^ As an emerging element in flexible electronic devices, organic field‐effect transistors (OFETs) have distinguished functions such as signal transduction and amplification.^14^, ^15^ At the same time, they are light in weight, flexible, and can be manufactured economically on plastic substrates by large‐area solution processing.^16^, ^17^ Benefitting from these salient features, OFETs are considered as excellent candidates for high‐performance flexible pressure sensors.

To achieve OFET‐based pressure sensors, many efforts have been devoted to designing device structures, optimizing dielectric/semiconductor materials, as well as tuning gate electrodes.^6^, ^18^, ^19^, ^20^, ^21^, ^22^, ^23^, ^24^ For instance, Someya et al. introduced OFET as a readout element for conductive rubber pressure sensors.^18^ By using microstructured polydimethylsiloxane (PDMS) as the dielectric, Bao and co‐workers developed active OFET sensors for pressure detection,^21^ and obtained a high sensitivity of 8.2 kPa^−1^ with fast response time.^6^ Recently, a suspended gate was incorporated into OFETs by Zhu and co‐workers, to realize ultrasensitive pressure sensors with a sensitivity as high as 192 kPa^−1^.^23^ These breakthroughs promise a bright future for OFET‐based pressure sensors. However, these OFET‐based sensors usually require high‐voltage operation, which has negative effects on the safety and low‐power consumption demands in wearable electronics. The high voltages used for these OFET‐based sensors are mainly due to the low capacitance gate dielectrics. It is known that the operating voltage of OFETs can be reduced by decreasing the thickness or increasing permittivity of gate dielectrics.^17^ To date, dielectric materials including self‐assembled monolayers, thin cross‐linked polymers, and high‐*k* metal oxides have been employed to reduce the operating voltages of OFETs.^25^, ^26^ Nevertheless, these thin organic dielectrics or high permittivity inorganic dielectrics are usually fragile for flexible devices, resulting in the increased gate leakage current and charge trapping at the semiconductor/dielectric interface.^27^, ^28^ Meanwhile, their relatively harsh and complicated fabrication processes prevent large‐scale manufacture of flexible OFETs.^25^ Recently, incorporation of thick polyelectrolytes as dielectrics was demonstrated to be an alternative method to fulfill high capacitance and thus low‐voltage operation of OFETs.^28^, ^29^, ^30^ Despite these interesting merits, polyelectrolyte materials have severe disadvantages of large leakage current, high hysteresis, and poor stability,^31^ which largely limit their utilizations in OFETs, particularly for the OFET‐based pressure sensors. Therefore, it is necessary to tailor polyelectrolyte dielectric layers to prevent leakage currents and improve operational stability, which are essential for low‐voltage OFETs and related sensors. Notably, a significant step to realize good e‐skin is to construct large‐area flexible pressure sensors, which require low‐voltage operation, high sensitivity in both low‐ and medium‐pressure regimes (0–10 and 10–100 kPa, respectively), short response time in the millisecond range, good flexibility, and stability for wearable device applications.^9^ However, flexible low‐voltage OFET‐based pressure sensors with high sensitivity have been seldom demonstrated so far, due to the absence of suitable gate dielectrics. Therefore, novel solution‐processable dielectric materials are highly desirable for the development of flexible and stable high‐sensitivity OFET‐based pressure sensors working at or below −5 V.

Herein, we demonstrate an effective and convenient strategy to develop bilayer polymer dielectrics for high‐performance flexible low‐voltage OFETs in pressure‐sensing applications. By using facile solution processing, thin poly(methyl methacrylate) (PMMA) with controlled thicknesses is able to combine with thick polyelectrolyte of polyacrylic acid (PAA) and thus generate a novel type of bilayer dielectrics. This multifunctional dielectric layer can provide a vertical phase separation structure with high capacitance, good stability, and remarkable reduction in leakage currents. As a result, the flexible OFETs using the PMMA/PAA dielectrics maintain low‐voltage operation and exhibit improved electrical performance than that of the devices with a pure PAA dielectric. Furthermore, we present the design and fabrication of flexible low‐voltage OFET‐based sensors which adopt a suspended gate and use an optimized PMMA/PAA dielectric layer to achieve highly sensitive pressure sensing. The OFET‐based pressure sensors reveal a record high sensitivity at a low operating voltage of −5 V, a fast response time of less than 20 ms, good flexibility, and bending stability. Our results provide a new viable way of dielectric engineering to develop flexible low‐voltage OFETs and related pressure sensors toward flexible electronic applications.

## 2 Results and Discussion

### 2.1 Device Structure and Materials for Flexible OFETs

**Figure 1:**
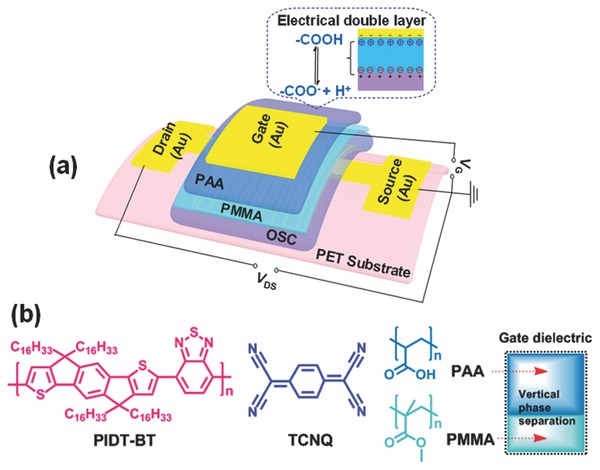
a) Schematic structure of the flexible OFET device. b) Molecular structures of PIDT‐BT, TCNQ, PAA, and PMMA (inset is an illustration of the PMMA/PAA dielectric with a vertical phase separation structure).

To achieve high‐performance flexible OFETs, interface engineering of dielectric materials on organic semiconductors for suppressing leakage currents and forming good interfacial contacts is critical to enhance the charge carrier transportation.^32^, ^33^ We chose a top‐gate/bottom‐contact device configuration to fabricate efficient and stable flexible OFETs. As schematically illustrated in **Figure** **1**a, the OFET device is composed of flexible substrate, source/drain (S/D) electrodes, organic semiconductor (OSC), dielectric, and gate electrode. Flexible polyethylene terephthalate (PET) was used as the bottom plastic substrate on which patterned gold (Au) was deposited to serve as the S/D electrodes. The conjugated polymer (PIDT‐BT) was doped with a small amount of TCNQ (≈2 wt%) as a small‐molecule additive to form an OSC layer. Their molecular structures are shown in Figure 1b. This solution‐processed polymer/small‐molecule composite can act as an effective active layer for enabling high environmental/operational stability and good uniformity of OFETs.^34^ As one of key elements of the OFETs, the gate dielectric was solution processed by combining a thick polyelectrolyte film of hydrophilic PAA with a thin insulator layer of lipophilic PMMA. The molecular structures of PAA and PMMA are also shown in Figure 1b, where the formed polymer composite dielectric layer has an inherently vertical phase separation structure (as illustrated in the inset of Figure 1b). Note that PAA is an attractive ion‐conducting polyelectrolyte dielectric showing high capacitance, which results from the formation of an electrical double layer (as presented in Figure 1a) due to ion charge separation upon an applied voltage to the gate.^31^ Although the high unit‐area capacitance of PAA dielectric allows it to induce high carrier densities in the channel under low‐voltage operation,^35^ its negative impacts such as high hysteresis, low switching speed, and large leakage current should be overcome for the OFET application. Herein, a thin PMMA layer with controllable thicknesses was incorporated between the OSC and PAA as a modified dielectric component to suppress leakage currents and protect the underlying OSC from atmospheric oxygen and moisture. Flexible PMMA is well known for its easy solution processability, good film formation, excellent dielectric properties, and outstanding stability.^36^ More importantly, the good interfacial compatibility of PMMA with OSC promotes the formation of a good contact at the semiconductor/dielectric interface, which is significant for the OFET performance in terms of suppressed hysteresis and improved stability. These attributes make the PMMA/PAA composite a promising multifunctional dielectric material for regulating and enhancing device performance of flexible low‐voltage OFETs and the resulting pressure sensors. To complete the fabrication of final OFETs, Au was thermally evaporated as the gate electrode on the top of the dielectric.

### 2.2 Electrical Performance of Flexible OFETs

**Figure 2:**
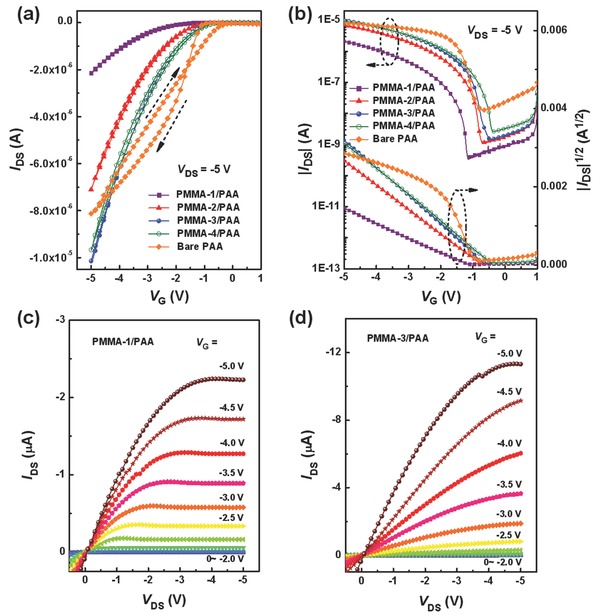
a,b) Transfer and c,d) output characteristics of the flexible OFETs using different PMMA/PAA dielectric layers.

To evaluate the applicability for flexible OFETs, several composite PMMA/PAA layers with controlled PMMA thicknesses were prepared as the gate dielectrics. Four polymer dielectric layers are labeled as PMMA‐1/PAA, PMMA‐2/PAA, PMMA‐3/PAA, and PMMA‐4/PAA for the PMMA thicknesses of ≈240, 60, 30, and 15 nm, respectively. For comparison, the control OFETs with bare PAA as a single dielectric layer were also fabricated. **Figure** **2** presents typical transfer and output characteristics of various flexible OFETs measured in ambient environment. Device parameters of these flexible OFETs extracted from the transfer characteristics are summarized in **Table** **1**. The field‐effect mobility (*µ*) was calculated from the saturation regime by the equation of *I*_DS_ = (*W*/2*L*)*C*_i_ *µ*(*V*_G_−*V*_T_)^2^, where *I*_DS_ is the source–drain current, *C*_i_ is the capacitance per unit area of the dielectric, *V*_G_ is the gate voltage, *V*_T_ is the threshold voltage, *W* and *L* are the channel width and length, respectively. As shown in Figure 2a, the transfer curves of the flexible OFETs based on the pure PAA dielectric show serious hysteresis. By contrast, all the flexible OFETs using the PMMA/PAA dielectrics exhibit significantly suppressed hysteresis for the transfer characteristics. All polymer composite dielectrics show a greatly reduced off current (Figure 2b), and the current‐deceasing factor reaches a maximum for the PMMA‐1/PAA. This great difference can be attributed to the fact that the off current is influenced by the electrical insulation of different polymer dielectric layers used. Interestingly, the on current is not suppressed by thin PMMA layers for the PMMA‐3/PAA and PMMA‐4/PAA dielectrics in the OFETs. The representative output characteristics display linear and saturation regimes in *I*_DS_ curves as the increase of negative *V*_G_ values, showing a p‐channel behavior for these flexible OFETs (Figure 2c,d). Moreover, the linear/saturation regimes correspondingly appearing at the lower/higher *V*_DS_ also suggest that the formation of an Ohmic contact between the S/D electrodes and the OSC layer.^32^

**Table 1:** Gate dielectric materials and device parameters of the flexible OFETs using different polymer dielectric layers

Dielectric material	PMMA thickness	*C*_i_ [nF cm^−2^]	*V*_T_ [V]	*µ*_max_ (*µ*_avg._) [cm^2^ V^−1^ s^−1^]	*I*_on_/*I*_off_ ratio
Bare PAA	0	13.11	−0.89	0.29 (0.21 ± 0.059)	10^3^
PMMA‐1/PAA	240	8.59	−1.24	0.23 (0.22 ± 0.016)	10^4^
PMMA‐2/PAA	60	9.04	−0.72	0.58 (0.57 ± 0.028)	10^4^
PMMA‐3/PAA	30	9.92	−0.61	0.69 (0.65 ± 0.038)	10^4^
PMMA‐4/PAA	15	10.81	−0.38	0.56 (0.51 ± 0.047)	10^4^

All the flexible OFETs using different PMMA/PAA dielectrics can work well with a low operating voltage of −5 V similar to that using the PAA dielectric. The reference OFETs with the pure PAA dielectric layer exhibit a *µ*_max_ of 0.29 cm^2^ V^−1^ s^−1^ with a *V*_T_ of −0.89 V and an on*/*off current ratio of ≈10^3^ (Table 1). By contrast, the average mobilities and on‐off current ratios of the flexible OFETs are simultaneously improved by using thin PMMA/PAA to replace bare PAA as the gate dielectric. Compared to the reference device, the incorporation of controllable PMMA layers leads to an obvious change in the threshold voltage of these flexible OFETs. It is found that the PMMA in composite dielectric layers plays an important role in affecting the OFET performances (Figure 2). Notably, the *V*_T_ of these flexible OFETs decreases from −1.24 to −0.38 V with the gradually reduced PMMA thickness from 240 to 15 nm. The on/off current ratios of these flexible devices using the PMMA/PAA dielectrics are around 10^4^. It is interesting that small variations (3.4–18.8%) in the *µ*_max_ are observed for the gate dielectrics with varying PMMA thicknesses from 15 to 60 nm. The mobility reaches a maximum value for the PMMA‐3/PAA when the PMMA thickness reaches 30 nm and drops drastically for the PMMA‐1/PAA with 240‐nm‐thick PMMA. With the decrease of PMMA thickness in the PMMA/PAA dielectrics, the OFETs exhibit enhanced mobility due to the increased capacitance, and the improved interface between PMMA and the organic semiconductor. However, ultrathin PMMA (e.g., 15 nm) might be too thin to efficiently improve the interface between PMMA and the semiconductor which would have large effects on the traps density as well as the leakage current. Therefore, the flexible OFETs based on the PMMA‐3/PAA dielectric (30 nm PMMA) afford the highest *µ*_max_ of 0.69 cm^2^ V^−1^ s^−1^, a *V*_T_ of −0.61 V, and an *I*_on_/*I*_off_ of 10^4^. Generally, the flexible OFETs using the PMMA/PAA dielectrics (with PMMA thicknesses of 15, 30, and 60 nm) are superior to those with the bare PAA dielectric regarding the overall electrical performance under low‐voltage operation. We further examined that this type of polymer composite dielectrics can be effective for the flexible OFETs under a further reduced operating voltage of −2 V. As displayed in Figure S1 (Supporting Information), the flexible device works well and exhibits good output and transfer characteristics, demonstrating that the PMMA‐3/PAA dielectric is promising for constructing flexible low‐voltage OFETs in pressure‐sensing applications.

**Figure 3:**
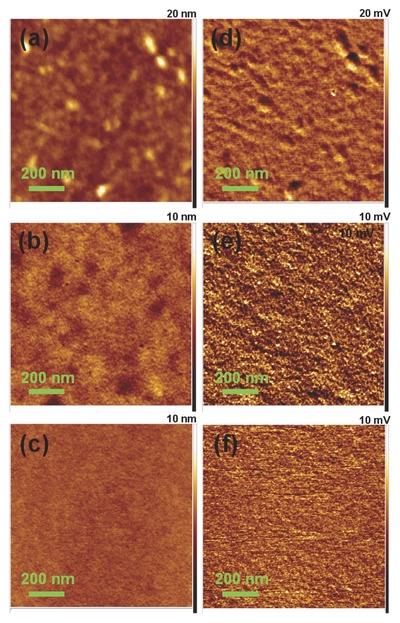
Tapping‐mode AFM analysis of a,d) OSC film, b,e) PMMA on OSC, and c,f) PAA on OSC/PMMA used in OFETs: a–c) height and d–f) adhesion images (1 × 1 µm).

The significant improvement in OFET performance is related to the vertical phase separation structure of the PMMA/PAA dielectric. We employed atomic force microscopy (AFM) to examine the changes in surface characteristics of the OSC layer of PIDT‐BT:TCNQ and the optimal dielectric of PMMA‐3/PAA for OFETs. Height and adhesion images of OSC layer, PMMA on OSC, and PAA on OSC/PMMA are shown in **Figure** **3**. A difference in the surface structure before and after depositing thin PMMA on the OSC is clearly observed as evidenced by a smaller size of nanocolloids on the surface of PMMA compared to a nanograin surface of the OSC (Figure 3a,b). Similarly, the thick PAA on OSC/PMMA also shows a very smooth surface structure (Figure 3c). The changes in polymer phase and phase size for PMMA and PAA in the dielectric are obvious from their adhesion images (Figure 3e,f), as differing from that of the OSC surface (Figure 3d). With gradual deposition of dielectrics on OSC, the surface of these solution‐processed films becomes smoother. Correspondingly, the root‐mean‐square (RMS) roughness of the surface decreases from 1.20 nm (OSC) to 0.58 nm (OSC/PMMA), and to 0.30 nm (OSC/PMMA/PAA), respectively. These results are in good agreement with the trend in topography AFM images of various films (Figure S2, Supporting Information). In this type of dielectrics, the achieved smooth and dense PMMA on top of the OSC layer is conducive to improve the interfacial contact between the OSC and the subsequent PAA to decrease leakage currents and thus promote charge transport in the channel. The lipophilic PMMA phase adjacent to the OSC layer will effectively protect and encapsulate the underlying OSC, and prevent the negative interaction on charge carriers from moisture.[[qv: 36c]] This is one of the main possible reasons for enhancing operational stability of the OFETs with the multifunctional PMMA/PAA dielectric. Meanwhile, the PAA polyelectrolyte in the composite dielectric offers a high electrical double‐layer capacitance which significantly increases the carrier concentration and accordingly improves the output current and carrier mobility. Compared to the pure PAA dielectric, this PMMA/PAA dielectric can provide good interfacial compatibility with the OSC layer while maintain a high capacitance, which are beneficial for reducing leakage currents and improving charge transport in the conductive channel. On the other hand, it is believed that an important feature of the PMMA/PAA dielectric layer is their accumulated barrier effect on atmospheric oxygen and moisture. Benefiting from this good vertical phase separation structure, the overall electrical performance and the operational stability of these flexible OFETs using the PMMA/PAA bilayer dielectrics are much better than those of the devices with the pure PAA dielectric.

**Figure 4:**
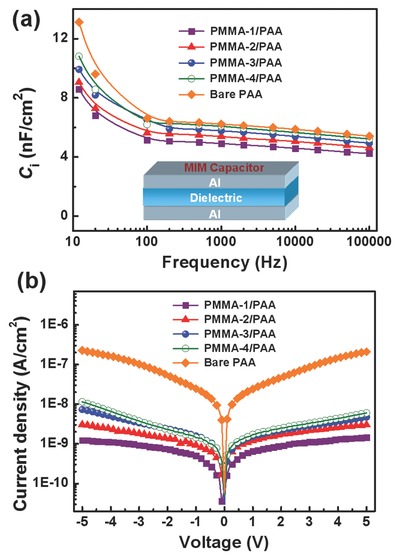
a) Capacitance–frequency characteristics of different polymer dielectric layers in a MIM capacitor (inset is a schematic device structure). b) Leakage current density–voltage characteristics of different polymer dielectric layers.

To further investigate the reasons why thin PMMA in the dielectrics has produced a significant effect on the electrical performance, we prepared several parallel capacitors using the different PMMA/PAA and PAA dielectrics. The device configuration is a metal–insulator–metal (MIM) structure of Al/dielectric/Al (see the inset of **Figure** **4**). As demonstrated in Figure 4a, after combination of the thin PMMA with the thick PAA, the dielectric capacitance mildly decreases across the entire low‐ and high‐frequency regions. With the increase of PMMA thickness in the PMMA/PAA dielectrics, the corresponding *C*_i_ values at 12 Hz of the MIM capacitors are determined to be 10.81, 9.92, 9.04, and 8.59 nF cm^−2^, respectively. Although they are less than that of bare PAA‐based MIM capacitor (13.11 nF cm^−2^), the capacitances of PMMA/PAA dielectrics remain at a high level to promote the low‐voltage operation for OFETs. Figure 4b further presents leakage current density–voltage characteristics of the MIM capacitors using different polymer dielectrics. It can be seen in the figure that the entire curves are nearly symmetric between the positive and negative biases. Importantly, the leakage current density of the only PAA dielectric has been greatly suppressed by adopting thin PMMA as a modified component in the PMMA/PAA dielectric layers. For the only PAA dielectric, a relatively high leakage current density is determined to be 2.25 × 10^−7^ A cm^−2^ at −5 V. By comparison, the PMMA/PAA dielectrics exhibit much lower leakage current densities of 1.22 × 10^−9^ to 1.14 × 10^−8^ A cm^−2^ at −5 V. With the increase of PMMA thickness, the leakage current density of PMMA/PAA dielectrics successively decreases, leading to a very low level of ≈10^−9^ A cm^−2^, which is decreased more than two orders of magnitude compared to that of the pure PAA dielectric. These results indicate that the combination of thin PMMA as the modified dielectric component with the PAA is very efficient in reducing leakage currents of the bare PAA dielectric. The contributions of high capacitances and greatly reduced leakage currents are thus the major reasons for enhancing device performance. Consequently, the flexible OFETs using the bilayer dielectrics exhibit average mobilities of 0.22, 0.57, 0.65, and 0.51 cm^2^ V^−1^ s^−1^ for PMMA‐1/PAA, PMMA‐2/PAA, PMMA‐3/PAA, and PMMA‐4/PAA, respectively. These average mobilities of the flexible OFETs using the PMMA/PAA dielectrics are also found to be higher than those of the reference devices with the pure PAA dielectric (*µ*_avg._ = 0.21 cm^2^ V^−1^ s^−1^) (Table 1), further demonstrating the significant enhancement of device performance by incorporating the multifunctional PMMA/PAA dielectrics. The performances are also better than those of the control OFETs with only PMMA dielectric which show an average mobility of 0.11 cm^2^ V^−1^ s^−1^ at a much higher operating voltage of −30 V. The representative transfer and output curves of the OFETs based on only PMMA dielectric are provided in Figure S3 (Supporting Information).

### 2.3 Device Design and Sensing Performance of Flexible Pressure Sensors

**Figure 5:**
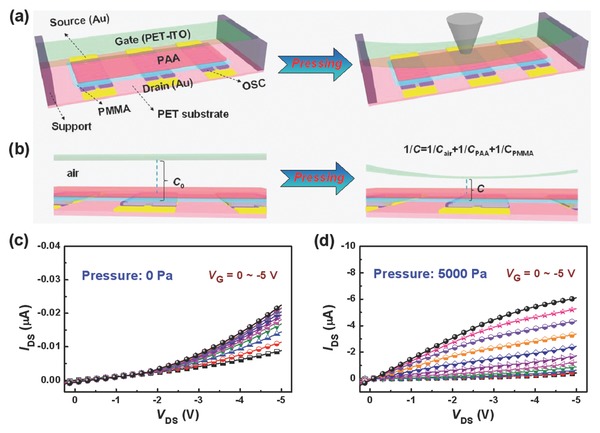
a) Schematic illustration of the flexible OFET‐based pressure sensor in the initial (left) and after pressing (right). b) Pressure‐sensing mechanism of the OFET sensor. c,d) Output characteristics of the OFET‐based pressure sensor measured at pressures of 0 and 5000 Pa, respectively.

As demonstrated above, the PMMA‐3/PAA layer is a promising dielectric material for realizing high‐performance flexible OFETs with the low operating voltages. Considering its balance in good dielectric performance, high capacitance, good robustness, and interfacial compatibility, it is expected that this new type of dielectrics can be used for low‐voltage high‐sensitivity OFET sensors. We thus further adopted the PMMA‐3/PAA as an efficient gate dielectric to develop flexible OFET‐based pressure sensors, as depicted in **Figure** **5**a. In this sensor, the patterned Au as bottom contact S/D electrodes, the OSC layer, and the PMMA‐3/PAA dielectric layer were sequentially fabricated on PET substrate in the same condition as that used for preparation of the above OFETs. Thin and flat tapes were then laminated onto the substrate as supports, which formed a large air gap space (≈110 µm). This gate‐absent device was then incorporated and fixed with a gate electrode of indium tin oxide (ITO)‐coated PET to complete the flexible suspended gate OFET‐based pressure sensors. Note that the PIDT‐BT film with TCNQ additive was still used as the OSC layer due to its good working stability as well as the low threshold voltage. Meanwhile, thin PET‐ITO was chosen as a suspended gate because of its good flexibility and stability, which enables deformation under pressing and recover back in shape after relaxation.

The pressure‐sensing mechanism of the sensor is illustrated in Figure 5b. Upon pressing, the air gap between the PET‐ITO gate and the PMMA‐3/PAA dielectric of the OFET sensor will reduce and cause an increase of the capacitance of air gap (*C*_air_). According to the fundamental of capacitance in series (1/*C* = 1/*C*_air_ + 1/*C*_PAA_ + 1/*C*_PMMA_), the total capacitance of gate dielectric in the suspended gate OFET sensor will correspondingly increase under the pressure. It has been demonstrated that a change in capacitance of the dielectric layer as a function of the applied pressure will be generated when the suspended gate deforms upon a force for both metal‐oxide‐semiconductor FETs and OFETs.^23^, ^37^ It is also known that the source–drain current is proportional to the capacitance density of gate dielectric.^38^ Therefore, when a force was applied to the suspended gate OFET sensor, the flexible PET‐ITO gate was deformed toward the PMMA‐3/PAA dielectric, resulting in the pressure‐dependent *I*_DS_ at a constant *V*_G_. The pressure‐sensing performance of this type of OFET sensors is strongly affected by the properties of the suspended gate and the dielectric, as well as the air gap space between the gate and the dielectric.^23^ A larger force leads to a smaller gap space and even a closer contact between the gate and dielectric, finally giving rise to an increased drain current of the designed pressure sensor. The key component of the sensor design is still the incorporated PMMA‐3/PAA dielectric. This dielectric layer has high capacitance and good dielectric performance to fulfill low‐voltage operation, low power consumption, and high reliability for the OFET sensor. As expected, such pressure‐sensitive device can be operated under a relative low‐voltage below −5 V, where its electrical response is further confirmed by pressure‐loaded output curves of the OFET‐based sensor. As displayed in Figure 5c, the initial device works at a low operating voltage of −5 V although its transfer characteristic is not typical, which may be due to the low capacitance and gate current leakage of the uncompressed air gap. By contrast, the compressed device at a pressure of 5000 Pa exhibits typical output transistor characteristics with good current amplification. The *I*_DS_ of compressed device at *V*_G_ = −5 V is increased by more than two orders of magnitude compared to that of the device without a pressure. It should be noted that the good saturation regime in the *I*_DS_–*V*_DS_ curves for the sensing device is not clearly observed. By further increasing the pressure, standard output characteristics with well‐defined linear and saturation regimes are observed (Figure S4, Supporting Information), which are similar to the OFETs using the PMMA‐3/PAA dielectric demonstrated in the previous section.

**Figure 6:**
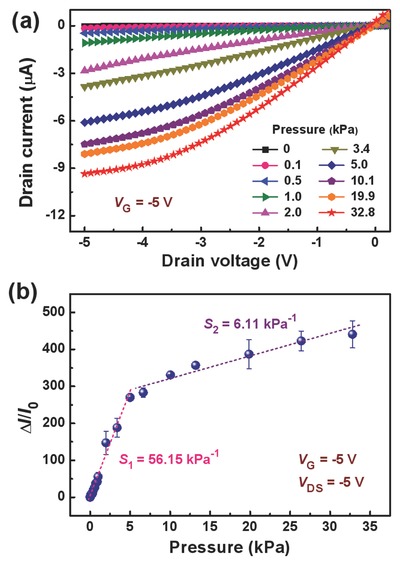
Electric characteristics of the flexible OFET‐based pressure sensor. a) Output curves of the sensor under different applied pressures at a constant *V*_G_ of −5 V, and b) relative change of source–drain current in response to the external pressure at constant *V*_G_ and *V*_DS_ of both −5 V.

The sensitivity (*S*) of our OFET‐based pressure sensor is defined as *S* = (Δ*I*/*I*_0_)/Δ*P*, where *I*_0_ is the initial current of the device without a loading pressure, Δ*I* and Δ*P* are the relative changes of current and applied pressure, respectively. To verify the pressure‐sensing responses, we investigated pressure‐dependent performance of the flexible OFET sensor. **Figure** **6**a presents several output curves of the OFET‐based pressure sensor under different applied pressures varying from 0 to 32.8 kPa. It can be clearly found that a linear and saturation current steadily rises as the successive increasing of applied pressure at *V*_DS_ of 0 to −5 V and a constant *V*_G_ of −5 V. This result agrees well with the working mechanism of the sensor elucidated above. The increment in drain current can be ascribed to the pressing on suspended PET‐ITO gate and thus causing a reduction in air‐gap space, which results in simultaneous enhancement of the capacitance and the gate electric field at the dielectric/OSC interface. Figure 6b further exhibits the source–drain current response to the external pressure when the OFET‐based sensor operated at constant *V*_DS_ and *V*_G_ of both −5 V. Notably, the pressure sensor exhibits high pressure sensitivity with a wide detection range, where different loading levels of external pressures for the device can be distinguished. In the low‐pressure regime, an average sensitivity (*S*_1_) up to 56.15 kPa^−1^ is achieved below 5 kPa. Above 5 kPa, the average sensitivity (*S*_2_) drops but still retains a high value of 6.11 kPa^−1^ in the medium‐pressure regime. The transition between the two sensitivity regions is mainly due to the pressure‐induced capacitance changes for the gate dielectrics of OFET sensors.^22^, ^23^ In the low‐pressure region, the capacitance increases drastically with a gradual reduction of air gap upon various external pressures. When the pressure reaches a threshold value (≈5 kPa), the flexible gate electrode starts to contact with the polymeric dielectric layer, and thus the capacitance is governed by the contact area between the two interfaces in the medium‐pressure region. The *I*_DS_ of OFET is directly correlated to the corresponding capacitance, therefore, our suspended gate OFET pressure sensors have two regions of sensitivity.

It is noteworthy that high sensitivities in both the low‐ and medium‐pressure regimes were obtained for the OFET‐based sensor at a low operating voltage of −5 V. The operating voltage is much lower than those of the previously OFET‐based pressure sensors reported so far.^6^, ^18^, ^19^, ^20^, ^21^, ^22^, ^23^, ^24^ By contrast, the control OFET sensors with a pure PMMA dielectric require a higher operating voltage of −10 V and deliver much lower sensitivities of 0.49 and 0.42 kPa^−1^ in the low‐ and medium‐pressure regimes, respectively (see Figure S5, Supporting Information). To further demonstrate that our OFET sensor can be operated at a much lower voltage for pressure sensing, we also measured the drain current response of the device at an operating voltage of as low as −2 V (Figure S6, Supporting Information). The achieved high sensitivities of the pressure sensor are 8.25 and 2.28 kPa^−1^ for the low‐ and medium‐pressure regimes, respectively. We compared our results with those of previously reported OFET‐based pressure sensors, as summarized in **Table** **2**. Notably, the operating voltage of our flexible OFET‐based pressure sensor is more than ten times lower than those of the reported OFET‐based pressure sensors using dielectric materials such as PDMS, poly(perfluorobutenylvinylether) (CYTOP), PMMA, polystyrene, and polyurethane.^6^, ^22^, ^23^ At the same time, the sensitivity of our pressure sensor can maintain a similar or even higher level. We also noted that the low operating voltage of our OFET sensors is close to that of the pressure sensors which were based on a thin‐film PDMS capacitive sensor connected with a floating‐gate OFET using a hybrid Al_2_O_3_/Parylene C dielectric.^39^ To the best of our knowledge, the achieved *S*_1_ of 56.15 kPa^−1^ is a record‐high sensitivity for low‐voltage OFET‐based pressure sensors to date. These results demonstrate that the multifunctional PMMA‐3/PAA is an efficient gate dielectric to enable low‐voltage and high‐sensitivity OFET‐based pressure sensors. Our designed sensors based on this bilayer dielectric have distinguished advantages of high pressure sensitivity, low‐voltage operation, and low power consumption, which offer a useful way to develop highly reliable and sensitive pressure sensors for wearable devices.

**Table 2:** Comparison on device performance of the OFET‐based pressure sensors in this work with those reported in the literatures

Dielectric material	Operating voltage [V]	Sensitivity [kPa^−1^]	Ref.
PDMS	−200	8.2	6
PMMA	−80	169.2	23
CYTOP	−60	192	23
Polystyrene	−60	121.2	23
Polyurethane	−50	1.76	22
Al_2_O_3_/Parylene Ca)	−2	n/a	39
PMMA‐3/PAA	−5	56.15	This work
PMMA‐3/PAA	−2	8.25	This work

^a)^ The pressure sensor was based on the floating‐gate OFET connected with a PDMS capacitive sensor.

**Figure 7:**
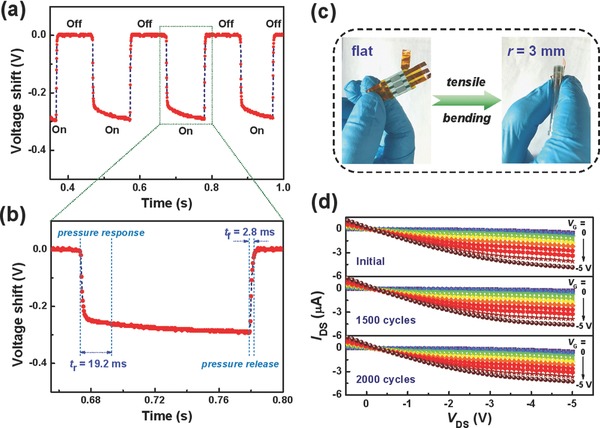
a) Cycle results and b) enlarged curve of one cycle for dynamic response of the low‐voltage OFET‐based pressure sensor upon loading/releasing an external pressure. c) Example of the flat OFET‐based pressure sensor bending into a convex shape. d) Output characteristics of the flexible OFET‐based pressure sensor under different bending cycles (*r* = 3 mm) at a low operating voltage of −5 V.

Besides the sensitivity, another significant parameter for sensing devices is response time. Thus, the dynamic response of our low‐voltage OFET‐based pressure sensor was further performed to assess its response time. We employed an oscilloscope to monitor the *I*_DS_ via a change in the voltage shift at both constant *V*_DS_ and *V*_G_ of −5 V, where an electrical equivalent circuit of the measurement system is provided in Figure S7 (Supporting Information). By repeated loading/releasing an applied pressure of 4 kPa, the cycle results of dynamic response of the sensor are presented in **Figure** **7**a. It is found that the pressure sensor shows a virtually immediate response process to an external force. From the enlarged figure of a single time‐resolved cycle, as shown in Figure 7b, the rise time (*t*_r_) of the response to a pressure loading is 19.2 ms, while the fall time (*t*_f_) of the response to a pressure releasing is 2.8 ms. The response time of the OFET‐based pressure sensor is comparable to that of the control sample with a pure PMMA dielectric (Figure S8, Supporting Information). These measured results reveal a rapid response of the low‐voltage OFET‐based sensor to an external force. This capability of fast response benefits from the virtues of the flexible and stable suspended gate of the OFET‐based pressure sensor.

Beyond the demands of high sensitivity and fast response time, good flexibility and bending stability of pressure sensors are also important for wearable electronic applications. Thus, we further studied the bending effect on device performance of the pressure sensor with the PMMA‐3/PAA dielectric. The sensor was bent perpendicular to the direction of source–drain current in the channel. Figure 7c shows an example of the flexible OFET‐based sensor deforming from a flat structure to a bended structure on a convex surface. The sensor can be bended from flat down to a small bending radius (*r*) of 3 mm and can be recovered well after bending, confirming its good flexibility. The high curvature is far more than demanded for wearable appliances, such as attaching the device to a human neck or wrist. To further verify bending stability of the low‐voltage OFET‐based pressure sensor, we investigated the impacts of bending cycles on the device performance. The flexible sensor was bent at *r* of 3 mm and the electrical characteristics were measured in ambient condition. Figure 7d shows output curves of the flexible low‐voltage OFET sensor during the repeated bending tests. Apparently, the sensor exhibits normal output characteristics without obvious discrepancy, demonstrating the stable OFET operation under different bending cycles. From the initial to 1500 cycles, the *I*_DS_ can maintain 95% of its initial value (shown in Figure S9, Supporting Information) for the flexible sensor at such a large bending (*r* = 3 mm), indicating the excellent stability of the PMMA/PAA dielectric and the OSC in the OFET sensor. With the bending cycle of 2000, the *I*_DS_ still retains more than 88% of the initial value which could be due to small changes within the dielectric/OSC layer of the device. The small degradation on the electrical performance over 2000 cycles demonstrates the high robustness, structure flexibility, and bending stability for the low‐voltage OFET‐based pressure sensor using this novel polymer bilayer dielectric. Additionally, we observed pressure‐sensitive output characteristics of the flexible low‐voltage OFET sensor under tensile bending (Figure S10, Supporting Information), which further demonstrate that this low‐voltage OFET sensor has the capability of pressure sensing in the bending state. Note that the OFET sensor was made on the 175‐µm‐thick PET substrate, which is still thick and thus limits the flexibility and bending performance. If a thinner substrate with better flexibility and stability is used for the highly flexible devices,^8^ the performance of these low‐voltage OFET‐based pressure sensor could be further improved.

All these results demonstrate that the flexible OFETs and pressure sensors using the novel bilayer PMMA/PAA dielectric provide a feasible approach to realize high‐performance flexible electronic devices. The achievements in low‐voltage operation, high pressure sensitivity, wide detection range, fast response time, and good flexibility and bending stability of the sensor indicate a bright future of low‐voltage OFETs for wearable pressure‐sensing applications. Therefore, the developed multifunctional dielectric materials as well as the low‐voltage OFETs and flexible pressure sensors are promising in broad applications such as e‐skin and soft robotics.

## 3 Conclusions

In summary, we have demonstrated a novel type of bilayer polymer dielectrics for achieving high‐performance low‐voltage OFETs and flexible pressure sensors. By combining a relatively thick PAA electrolyte layer with a thin PMMA layer to form a vertical phase separation structure, several composite PMMA/PAA dielectrics were obtained, delivering greatly reduced leakage currents while maintaining high capacitances. The fabricated flexible OFETs using these dielectrics enabled low‐voltage operation and exhibited greatly suppressed hysteresis and increased mobility compared to those with the pure PAA dielectric. Furthermore, flexible suspended gate OFET‐based pressure sensors have been developed based on the optimized PMMA/PAA dielectric, achieving a record high sensitivity of 56.15 kPa^−1^ at the operation of −5 V, a fast response time of less than 20 ms, and good flexibility with bending stability over 2000 cycles under a bending radius of 3 mm. Our results provide a convenient and efficient pathway to develop flexible low‐voltage OFETs and high‐sensitivity pressure sensors for practical e‐skin applications. These findings also indicate that this type of solution‐processable bilayer dielectrics shows great potentials in high‐safety and low‐power flexible optoelectronic devices, e‐skins, and portable or wearable electronics.

## 4 Experimental Section

*Materials*: Insulating PAA (*M*_v_ ≈450 000) and PMMA (*M*_w_ ≈120 000) were purchased from Sigma‐Aldrich Inc. and used to fabricate the gate dielectric layers. Semiconducting PIDT‐BT and TCNQ purchased, respectively, from Derthon Optoelectronic Materials Science Technology Co., Ltd. and Beijing J&K Scientific Ltd. were used to prepare the active OSC layer. Chlorobenzene (99.8%), butyl acetate (99%), and methanol (99.9%) were obtained from Sigma‐Aldrich Inc. and used as received. Other reagents and raw materials were provided commercially by Sigma‐Aldrich Inc. or Adamas‐beta Ltd., and used without further purification.

*Fabrication of Flexible OFETs*: All flexible OFETs were fabricated on PET with a thickness of 175 µm. After cleaning the PET substrate, Au source–drain electrodes (50 nm) were deposited on the PET by thermal evaporation through a shadow mask with the channel width (*W*) and length (*L*) of 22.5 mm and 0.15 mm, respectively. Prior to the preparation of OSC, the PET substrate with Au source–drain electrodes was treated with UV–O_3_ for 15 min and then transferred into a nitrogen‐filled glove box. Then, the OSC layer was fabricated by spin‐coating (2000 rpm, 60 s) a blend solution of PIDT‐BT:TCNQ on the PET/Au surface, followed by annealing at 80 °C for 20 min. The PIDT‐BT and TCNQ with a 98:2 w/w ratio were dissolved together in chlorobenzene to give a 5 mg mL^−1^ solution under string for a few hours at 40 °C. Subsequently, thin PMMA with controllable thicknesses was fabricated on the semiconductor by spin‐casting a 10 mg mL^−1^ of PMMA solution in butyl acetate. After storage for 30 min, a 30 mg mL^−1^ of PAA methanol solution was spin‐coated (500 rpm, 60 s) on the PMMA to form a thick PAA film (≈1 µm). Then the combined PMMA/PAA layer was thermally treated at 80 °C for 20 min to act as a composite dielectric layer. Finally, 50 nm thick Au as the gate electrode was deposited on top of the polymer dielectric by vacuum evaporation via a shadow mask to finish the OFET fabrication. All evaporation processes were performed under a high vacuum of ≈2 × 10^−4^ Pa and the evaporation rate for Au was maintained at around 0.5 Å s^−1^.

*Fabrication of Flexible Pressure Sensors*: To fabricate flexible OFET‐based pressure sensors, the Au source–drain electrodes were deposited on the PET substrate and the OSC layer was prepared as described above. Then, an optimized PMMA layer (≈30 nm) was used to combine with a thick PAA layer (≈1 µm) as the composite dielectric, which were fabricated the same as the conditions used for the OFETs. Thereafter, strips of tapes with 110 µm in thickness were laminated onto the substrates as supports. Finally, a 50‐µm‐thick PET film coated by ITO (≈6 Ω square^−1^, 185 nm in thickness) was transferred and fixed onto the support layer with tapes to act as the suspended gate.

*Fabrication of Parallel Capacitors*: Parallel capacitors were based on a MIM device configuration, which was comprised of two parallel metal plates with different polymer dielectric layers between the plates. In detail, two Al (100 nm) films were thermally evaporated as the bottom and top plate electrodes, respectively, through a specific shadow mask under a high vacuum (≈2 × 10^−4^ Pa). Between the two metal plates of Al electrodes, the different polymer dielectrics were fabricated under the same condition as that used for the OFETs. All the active device area for MIM capacitors was fixed at 7.07 mm^2^.

*Characterization*: Surface characteristics of different films were characterized by AFM (Bruker Dimension ICON). Film thicknesses and the gap space were determined by a Bruker Dektak XT surface profiler. Electrical characteristics of all flexible OFETs and OFET‐based pressure sensors were measured in ambient environment by using an Agilent 4155C semiconductor parameter analyzer connected to a probe station. Capacitance measurements of parallel capacitors were performed by TH2827C Precision LCR Meter. For the measurement of pressure responses, the force applied on the sensors was precisely controlled by a specially assembled force equipment. The contact area between the pressing tip and the sensors was fixed at 1.5 cm^2^, which was used to calculate the applied pressure. An oscilloscope (Tektronix TBS1052B), connected to the OFET‐based pressure senor, was utilized to measure the response time of the sensor. The source–drain current of OFET sensor was amplified through a current amplifier and transferred to be the voltage, which was further amplified via a precision instrumentation amplifier. The response signal was recorded by measuring the divided voltage of the OFET‐based pressure sensor which was connected with a 5 MΩ resistor in series.

## Conflict of Interest

The authors declare no conflict of interest.

## Supporting information

Supplementary
