# Supplementary material for: Solution‐Processed Bilayer Dielectrics for Flexible Low‐Voltage Organic Field‐Effect Transistors in Pressure‐Sensing Applications
Source: Adv Sci (Weinh). 2018 Jul 11;5(9):1701041. doi: 10.1002/advs.201701041 (PMC6145259; doi:10.1002/advs.201701041)
Supplement: Supplementary file 1 — Supplementary [file ADVS-5-1701041-s001.pdf]

Copyright WILEY-VCH Verlag GmbH & Co. KGaA, 69469 Weinheim, Germany, 2018.

ADVANCED SCIENCE
Open Access

# Supporting Information

for *Adv. Sci.,* DOI: 10.1002/advs.201701041

# Solution-Processed Bilayer Dielectrics for Flexible Low-Voltage Organic Field-Effect Transistors in Pressure-Sensing Applications

*Zhigang Yin, Ming-Jie Yin, Ziyang Liu, Yangxi Zhang, A. Ping Zhang,* and Qingdong Zheng**

WILEY-VCH

Copyright WILEY-VCH Verlag GmbH & Co. KGaA, 69469 Weinheim, Germany, 2016.

# Supporting Information

## Solution-Processed Bilayer Dielectrics for Flexible Low-Voltage Organic Field-Effect Transistors in Pressure Sensing Applications

*Zhigang Yin, Ming-jie Yin, Ziyang Liu, Yangxi Zhang, A. Ping Zhang*, and Qingdong Zheng**

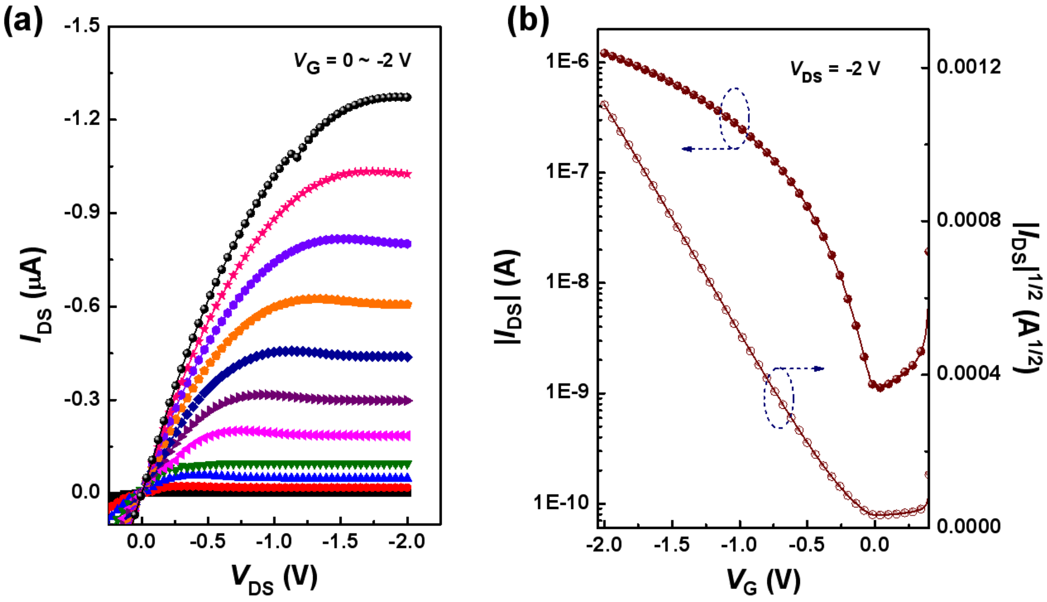


**Figure S1.** Output (a) and transfer (b) characteristics of the flexible OFETs using the PMMA-3/PAA dielectric under a low operating voltage of -2 V.

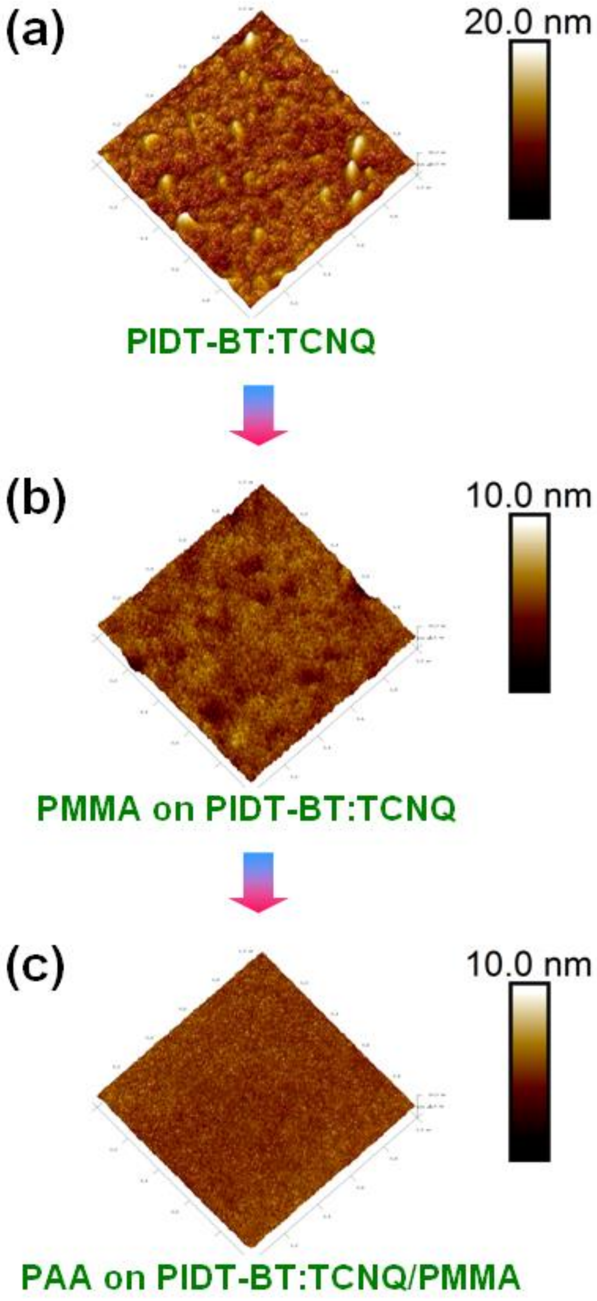


**Figure S2.** Topography AFM images (1×1 μm) of various films: (a) OSC, (b) PMMA on OSC, and (c) PAA on OSC/PMMA.

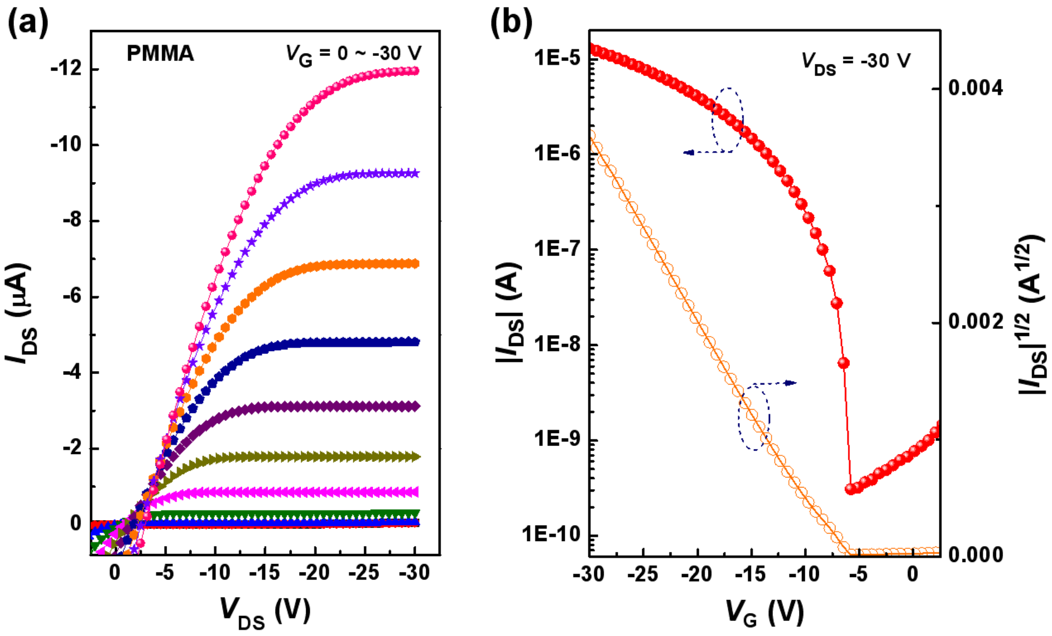


**Figure S3.** Output (a) and transfer (b) characteristics of the flexible OFETs using the only PMMA dielectric.

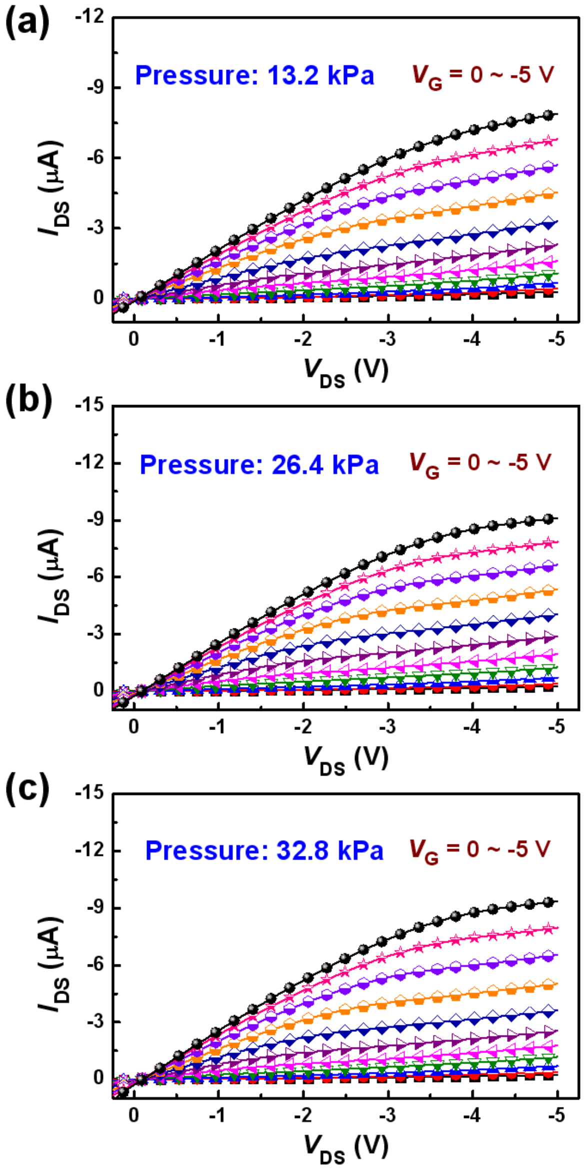


**Figure S4.** Output characteristics of the flexible OFET-based pressure sensor measured at different pressures of 13.2 kPa (a), 26.4 kPa (b), and 32.8 kPa (c), respectively.

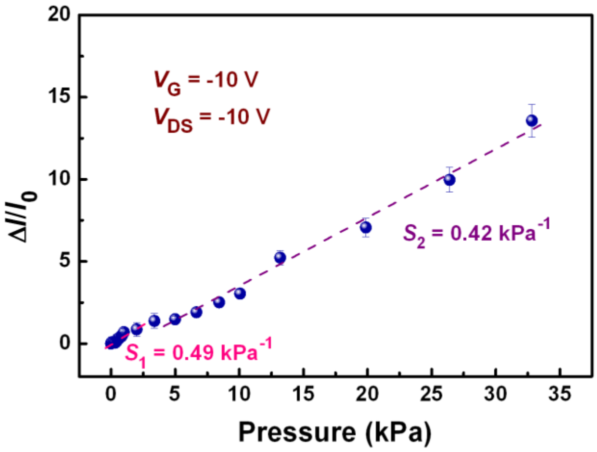


**Figure S5.** Relative change of the source-drain current in response to the external pressure for the control OFET-based pressure sensor with a pure PMMA dielectric at both constant $V_G$ and $V_{DS}$ of -10 V.

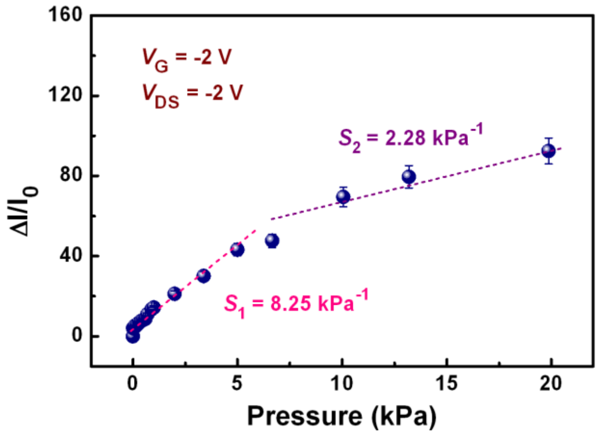


**Figure S6.** Relative change of the source-drain current in response to the external pressure of the flexible OFET-based pressure sensor at constant $V_G$ and $V_{DS}$ of both -2 V.

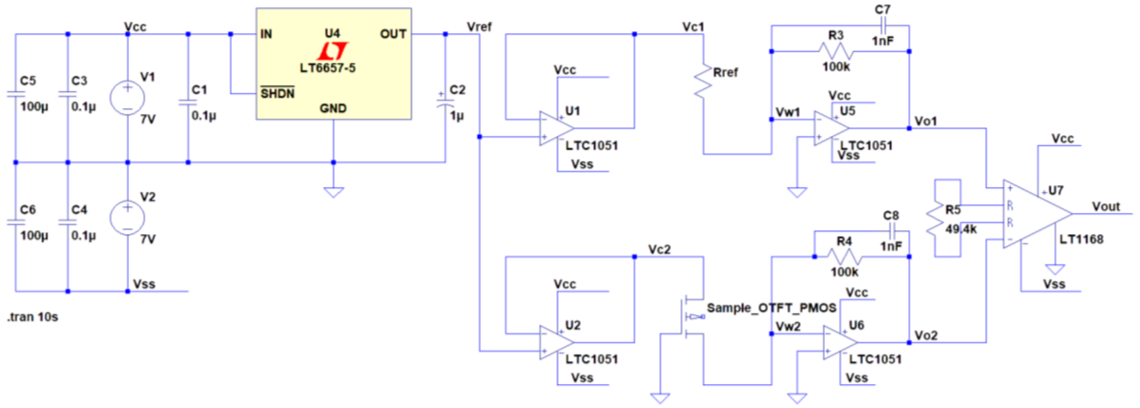


**Figure S7.** Electrical equivalent circuit of the measurement system for response time of the flexible low-voltage OFET-based pressure sensor.

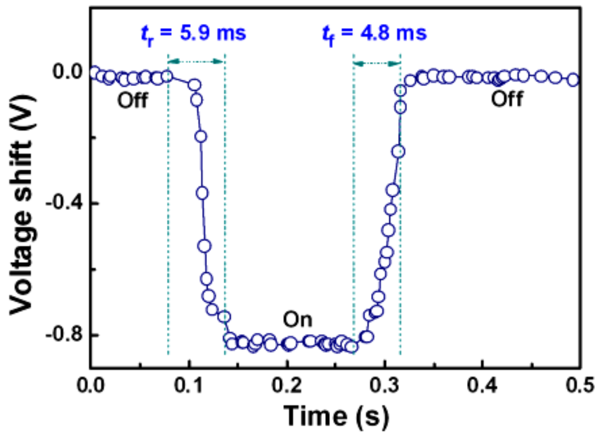


**Figure S8.** Oscilloscope recorded time-resolved response of the control OFET pressure sensor with a pure PMMA dielectric upon loading/releasing an external pressure.

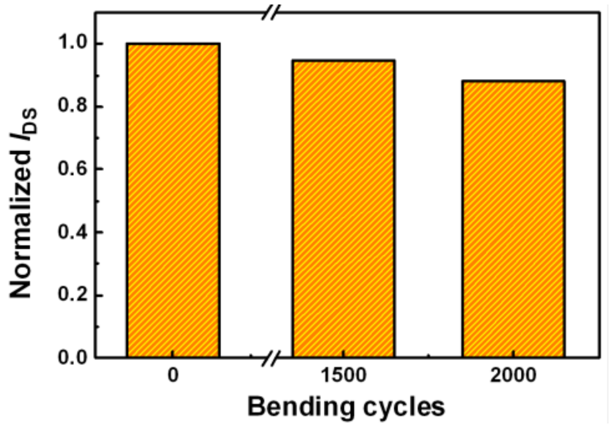


**Figure S9.** Change of the normalized $I_{DS}$ at both $V_G$ and $V_{DS}$ of -5 V for the flexible OFET-based pressure sensor under different bending cycles ($r$ = 3 mm).

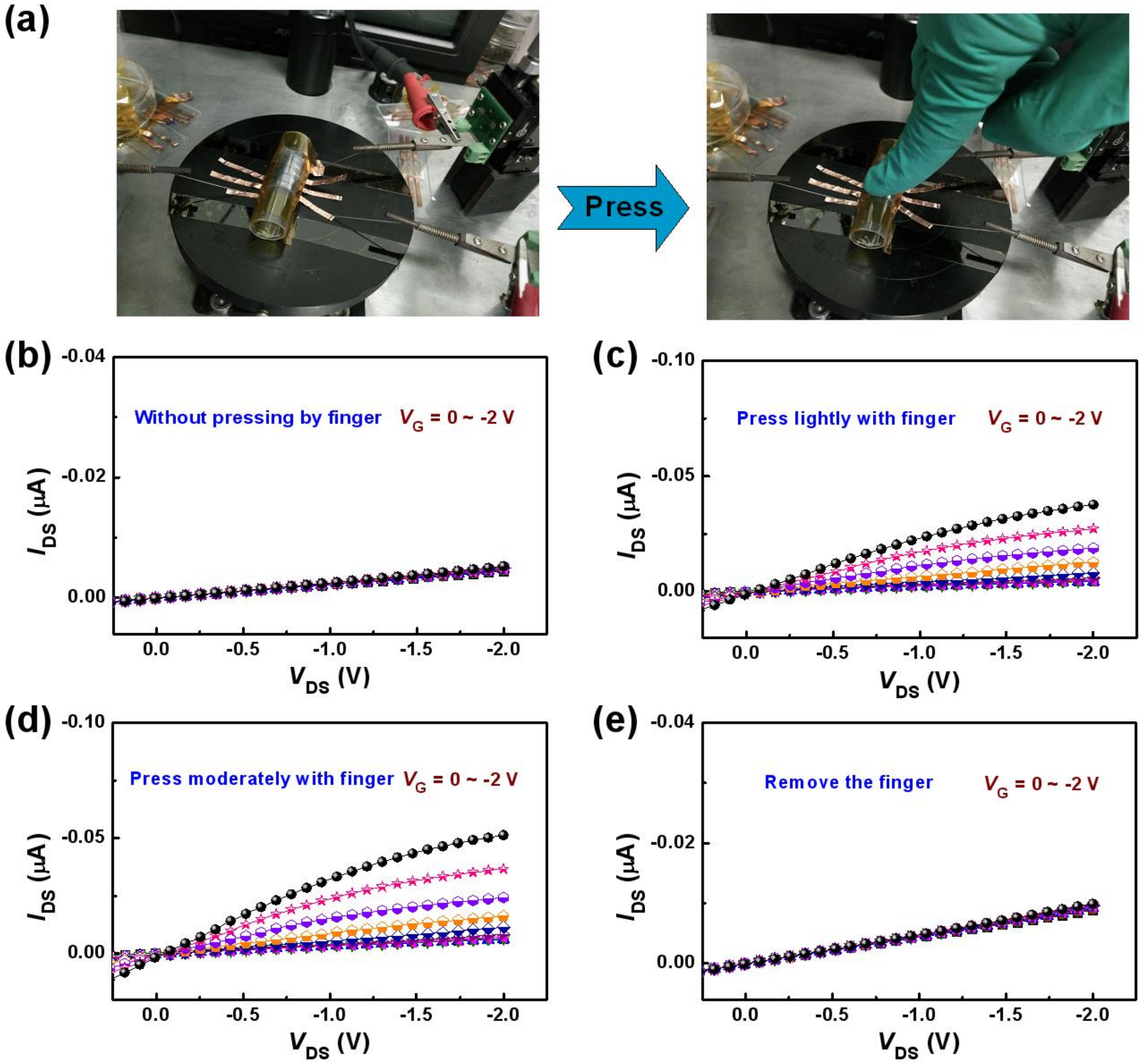


**Figure S10.** (a) Photographs of the flexible OFET-based pressure sensor in the bending state ($r$ = 7 mm) for pressure sensing. (b-e) Output characteristics of the flexible low-voltage OFET sensor measured at different conditions: (b) without pressing by finger, (c) press lightly with finger, (d) press moderately with finger, and (e) remove the finger.
